# Incidence and Time Trends of Type 2 Diabetes Mellitus among Adults in Zhejiang Province, China, 2007-2017

**DOI:** 10.1155/2020/2597953

**Published:** 2020-01-19

**Authors:** Meng Wang, Wei-Wei Gong, Jin Pan, Fang-Rong Fei, Hao Wang, Min Yu, Xiao-Yan Zhou, Ru-Ying Hu

**Affiliations:** Zhejiang Provincial Center for Disease Control and Prevention, 3399 Binsheng Road, Hangzhou 310051, China

## Abstract

**Aims:**

Population-based incidence data are paramount to assess the effects of prevention strategies for diabetes, yet the relevant studies in mainland China are scarce. This study is aimed at estimating the type 2 diabetes mellitus (T2DM) incidence and time trends in Chinese adults.

**Material and Methods:**

Based on the Diabetes Surveillance System of Zhejiang Province, 879,769 newly diagnosed T2DM cases were identified from January 1, 2007, to December 31, 2017. Annual incidence, incidence rate ratios (IRRs), and average annual percentage change with their 95% confidence intervals (CIs) were reported.

**Results:**

The age-standardized overall incidence rate of T2DM was reported to be 281.73 (95% CI: 281.26-282.20) per 100,000 person-years, 293.19 (95% CI: 292.51-293.87) in males and 270.42 (95% CI: 269.76-271.09) in females. Compared with the ≥80 years age group, younger adults were at lower risk for T2DM (IRRs ranged from 0.035 to 0.986 and the 95% CIs did not include the null), except for the 70-79 years age group (IRR: 1.087, 95% CI: 1.077-1.097). Compared with females and rural areas, the risk for T2DM was higher in males (IRR: 1.083, 95% CI: 1.079-1.088) and urban areas (IRR: 1.005, 95% CI: 1.001-1.009), respectively. The standardized annual incidence rate increased from 164.85 in 2007 to 268.65 per 100,000 person-years in 2017, with an average annual increase of 4.01% and grew more rapidly in male, younger, and rural area populations.

**Conclusions:**

Our study suggested a significant increase in the incidence rate of T2DM among Chinese over the past decade, especially in adults characterized by male sex, younger age, and rural areas.

## 1. Introduction

Diabetes has been a growing global problem. Reported by International Diabetes Federation, the diabetes prevalence among adults aged 20-79 years was 4.6% in 2000, which has risen to 8.8% in 2017 worldwide [1]. China is the largest developing country and has a huge population characterized by aging and urbanization. With rapid economic development, the diabetes epidemic in China has been continuing to increase. Two successive national surveys conducted in 2007 and 2010 showed that the prevalence of diabetes in Chinese residents was 9.7% and 11.6%, accounting for 92.4 million and 113.9 million adults with diabetes, respectively, [2–3]. However, little is known about the trends in diabetes incidence in China. Previous literature has declared that the indicator of incidence is more sensitive than prevalence when evaluating the time trends in chronic diseases, such as diabetes. This is because the prevalence depends on incidence and survival, and prevalence of diabetes could rise even if incidence does not, due to the improved survival. Besides, understanding the incidence of diabetes and identifying factors that contribute to the rising or falling trend in diabetes over time may better help target intervention measures. In recent years, the diabetes incidence rates and time trends in Chinese adults have been evaluated in several cohort studies [4–6]. However, these studies on diabetes incidence rates and time trends have some limits: firstly, the cohort studies usually only provide incidence rate during the follow-up period and it is difficult to examine the specific temporal trend year by year; secondly, the incident cases of diabetes are derived from a fixed cohort itself, not from a dynamic population. In the recent decade, population-based estimation of diabetes incidence and time trends among Chinese adults has also been attempted. Using diabetes registry data, a study in Harbin city with 26,953 new T2DM cases indicated that the Chinese population has experienced a rapid increase in the incidence of T2DM at an annual rate of 12% during 1999 and 2005 [7]. Based on electronic health records in Hong Kong from 2006 to 2014, a recent study found that diabetes incidence in Hong Kong Chinese appeared to have stabilized and there have been small declines during the study period [8]. However, considering the limited sample sizes, as well as the study period and regional representation in Harbin and Hong Kong, an updated study on the T2DM incidence among the Chinese population is warranted. The primary objective of the study is to examine the T2DM incidence and time trends in Chinese adults in Zhejiang province during the period 2007-2017 by age, sex, residence area, and calendar year.

## 2. Material and Methods

### 2.1. Data Sources

Data analyzed in the present prospective study was obtained from the Diabetes Surveillance System of Zhejiang Province, which was a population-based diabetes registry system maintained by Zhejiang CDC. The system was established in 2001 with 30 representative surveillance districts and over 16 million residents in the beginning. Up to 2009, diabetes registration based on the surveillance system has extended to all 90 districts throughout the province, covering approximately 48 million residents. The surveillance procedures and quality control measures have been described elsewhere [9–10] and are thus only briefly recounted here. All the diabetes cases were diagnosed by certificated health practitioners in local hospitals and health services centers. After the diagnosis was finished, the patients' information regarding demographics, diagnosis, and laboratory indicators were registered in the system within a week. To make sure that only the newly diagnosed cases were recorded, the patients registered in the system were further verified according to the characteristics of identity card number as well as the full name, gender, date of birth (year and month), and region code. Later, the confirmed and recorded patients would be followed-up once per year by the health practitioners in local health services centers. Furthermore, type 1, type 2, gestational, or other types of diabetes patients were registered in the system, and the classification of diabetes type and registration was completed by a health practitioner. All the recorded diabetes cases were coded according to the International Classification of Disease 10^th^ revision (ICD-10). This study was carried out in accordance with the “Declaration of Helsinki”, and the informed consents were obtained from cases and approved by the Ethics Committee of Zhejiang CDC.

### 2.2. T2DM Case Diagnosis

In the current study, T2DM cases were diagnosed using the following critical values according to World Health Organization (WHO) criteria [11]: (1) random plasma glucose ≥ 11.1 mmol/L, (2) fasting plasma glucose ≥ 7.0 mmol/L, or (3) 2 h plasma glucose value after the oral glucose tolerance test ≥ 11.1 mmol/L and presented classic symptoms. Whether the cases frequently had ketoacidosis at presentation or were required ongoing insulin therapy was used for the differential diagnosis of T2DM. Besides, serology examinations like beta-cell auto-antibodies and C-peptide were also conducted.

### 2.3. Population Size

The population size in each year was defined as the total population in a year in person-years. The total population data in each year were obtained from the Zhejiang Provincial Statistics Bureau and were calculated with the resident number estimated at the beginning and end of each year (i.e., midyear population).

### 2.4. Statistical Analysis

Descriptive statistics were used to describe the baseline characteristics of T2DM cases included in the analyses with frequency and proportion. The crude incidence rates were calculated as the number of T2DM cases divided by the population size. Considering the data accessibility and stability, the data involving 30 representative surveillance districts were used in the calculation from 2007 to 2014, and thereafter, we used the whole data throughout the province. The incidence was calculated for each age group (20-29, 30-39, 40-49, 50-59, 60-69, 70-79, and ≥80 years) and calendar year (2007-2017), stratified by sex (males and females). The standardized incidence was calculated using the direct standardization method according to the sixth population census in Zhejiang, 2010. To explore the effects of diagnosis year, age, sex, and residence area on incidence, Poisson regression models were conducted with reporting of the incidence rate ratio (IRR) and 95% confidence intervals (CIs). Within the model, the calendar year was treated as a dummy variable. To examine the time trends of T2DM incidence, the average annual percentage change in incidence was calculated in a multivariable Poisson regression model. Meanwhile, within the model, the calendar year was treated as a continuous variable and the statistical significance of the regression coefficient was tested. Interactions between calendar year and other covariates (sex and residence area) were also tested. All analyses were performed using SAS statistical package (version 9.2, SAS Institute, Inc., Cary, NC, USA).

## 3. Results

A total of 879,769 T2DM cases aged 20 years or more diagnosed between January 1, 2007, and December 31, 2017, were identified in this study. The mean age at diagnosis was 59.61 ± 13.23 years. The detailed baseline characteristics of T2DM cases included in the study are described in Table 1.

### 3.1. Sex and Residence Area

Between 2007 and 2017, the crude overall incidence rate of T2DM was 402.37 per 100,000 person-years (95% CI: 401.53-403.21). Standardized overall incidence rate of T2DM during the same period was estimated at 281.73 per 100,000 person-years (95% CI: 281.26-282.20), 293.19 (95% CI: 292.51-293.87) in males and 270.42 (95% CI: 269.76-271.09) in females (Table 2). In urban and rural areas, the crude incidence rate of diabetes was 410.93 per 100,000 person-years and 397.64 per 100,000 person-years, respectively (data not shown). After adjusting for other covariates in the Poisson regression models, the risk for T2DM was 1.083 times (IRR: 1.083, 95% CI: 1.079-1.088) and 1.005 times (IRR: 1.005, 95% CI: 1.001-1.009) higher in males and urban areas, respectively (Table 3).

### 3.2. Age Groups

The mean incidence rate was significantly different across all age groups in both males and females, ranged from 34.44 per 100,000 person-years (95% CI: 33.62-35.27) to 994.03 person-years (95% CI: 982.83-1005.32) in males and 28.89 per 100,000 person-years (95% CI: 28.14-29.66) to 1029.38 per 100,000 person-years (95% CI: 1022.06-1036.74) in females. The highest incidence rate was seen in the 70-79 years age group, followed by the ≥80, 60-69, 50-59, 40-49, 30-39, and 20-29 years age groups (Table 2). Compared with the ≥80 years age group, adults aged 70-79 years were at significantly higher risk for T2DM (IRR: 1.087, 95% CI: 1.077-1.097), while the 60-69, 50-59, 40-49, 30-39, and 20-29 years age groups were at significantly lower risk for T2DM, with IRR of 0.986 (95% CI: 0.977-0.994), 0.688 (95% CI: 0.682-0.694), 0.316 (95% CI: 0.313-0.319), 0.113 (95% CI: 0.112-0.115), and 0.035 (95% CI: 0.035-0.036), respectively (Table 3).

### 3.3. Incidence Time Trends

**Figure 1:**
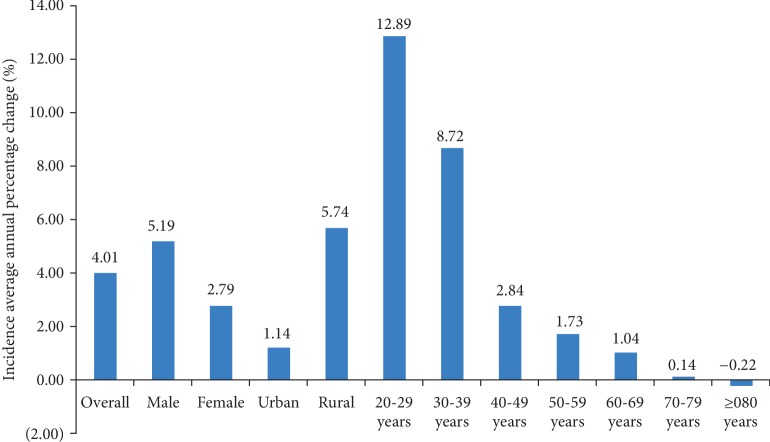
Average annual percentage change of type 2 diabetes incidence by demographic factors.

Annual incidence rate and average annual percentage changes of incidence are shown in Table 4 and Figure 1. The specific data of average annual percentage changes of incidence are shown in Supplementary [Supplementary-material supplementary-material-1]. The standardized annual incidence rate increased from 164.85 per 100,000 person-years in 2007 to 268.65 per 100,000 person-years in 2017. Specifically, incidence rate rose from 2007 (164.85 per 100,000 person-years) to peak at 2014 (346.52 per 100,000 person-years) and subsequently declined through to 2017 (268.65 per 100,000 person-years). For the adults aged 20 years or more, a statistically significant increase in incidence was seen, with an average annual increase of 4.01% (95% CI: 3.94-4.08). Besides, the average annual percentage change decreased with age and it was greater in males (5.19%, 95% CI: 5.09%-5.30%) than in females (2.79%, 95% CI: 2.69%-2.89%). For residence area, the average annual incidence increased by 5.74% (95% CI: 5.64%-5.83%) in rural areas, which was higher than that in urban areas (1.17%, 95% CI: 1.06%-1.28%). Finally, no statistically significant interactions were observed between calendar year and sex or residence area.

## 4. Discussion

With the population-based register of people with diabetes in Zhejiang province of China, we have reported the incidence and time trends of T2DM among adults between 2007 and 2017 and examined the effects of age, sex, and residence area on these estimates. Overall, our findings suggested that the incidence of T2DM increased significantly from 2007 to 2017, and differences in incidences and time trends were also observed by age, sex, and residence area. Over the last few years, we also conducted a similar study among youth with T2DM registered in the diabetes registry system. Consistently, during the study period 2007-2013, an alarming increase in T2DM incidence in children and adolescents was observed [9].

During 2007-2017, the age-standardized overall incidence rate of T2DM was reported to be 281.73 per 100,000 person-years (293.19 in males and 270.42 in females) among adults aged 20 years or more. Our estimates were higher than those in Netherlands population aged ≥19 years between 1998 and 2000 (the age and sex-adjusted incidence per 100,000 person-years was 227 overall, for men 222 and for women 231) [12] but lower than other results in previous literature. Based on new diabetes cases registered from 1972 to 2001, a Swedish study found that the age-standardized incidence rate for T2DM was 303 per 100,000 person-years in rural residents aged 35 to 79 years [13]. Besides, a register-based study in Scotland between 2004 and 2013 reported that the age-standardized overall incidence rate of T2DM was 403 per 100,000 person-years (488 in men and 333 in women) in people over 39 years of age [14]. More recently, using the database of Clinical Practice Research Datalink, another UK-based study showed that the standardized incidence rate of T2DM was 507.7 and 361.0 per 100,000 person-years for men and women aged ≥16 years during 2004 and 2014 [15]. With participants (baseline age 45-74 years) from five regional population-based studies conducted between 1997 and 2010, the Diabetes Collaborative Research of Epidemiologic Studies (DIAB-CORE) consortium reported that the T2DM incidence rate in Germany was 1180 per 100,000 person-years [16]. The variation in the reported incidence rates in different countries could theoretically be due to the differences in T2DM diagnosis, study designs, population characteristics, study periods, and statistical methods.

In our study, the age-standardized annual incidence rate increased by 62.97%, from 164.85 per 100,000 person-years in 2007 to 268.65 per 100,000 person-years in 2017, which was consistent with the increasing prevalence of diabetes among Chinese adults between 2007 and 2013 [2–3, 17]. Meanwhile, our results were comparable to the findings from other worldwide populations in different time periods. Using the Health Improvement Network database, a UK study indicated that the incidence rate of T2DM rose from 260 per 100,000 person-years to 431 per 100,000 person-years over the period between 1996 and 2005, which represented an increase of 65.77% [18]. Data from the National Health Interview Survey in the USA suggested a 36.54% increase in age-adjusted incidence rate of diabetes among adults aged 18 to 79 years, from 520 per 100,000 population in 1997 to 710 per 100,000 population in 2003 [19]. Furthermore, the average annual percentage change in rate of the incidence of T2DM was reported to be 4.01%, which was lower than the result (12%) from a Chinese study in Harbin (1999-2005) [7] but similar to the annual increase of 4.70% in diabetes incidence among adults aged 20 to 79 years in the USA during 1990-2008 [20]. In Stockholm inhabitants aged ≥18 years, a study showed that the incidence of diabetes rose at an annual rate of 3.0% between 1990 and 2002 [21]. Data from the National Danish Diabetes Register also indicated that the incidence rate of diabetes increased about 5% per year over a 10-year period (1995-2006) [22]. Although reasons for the rising incidence of T2DM were complicated and uncertain, we speculated that increasing trends of prediabetes- and diabetes-related factors as well as a declining missing report rate of the diabetes surveillance system may be responsible. Nationally representative surveys have indicated that the prevalence of prediabetes in Chinese adults rose from 15.5% in 2007 to 35.7% in 2013 [2–3, 17]. Based on previous reports [18, 21, 23], an appreciable portion of the rise in incidence could be attributed to the increased body mass index (BMI) during the observation periods. A series of national surveys showed that the proportion of Chinese adults who were obese increased from 8.6% in 2000 to 10.3% in 2005, 12.2% in 2010, and 12.9% in 2014 [ 24]. In Zhejiang province of China, the standardized prevalence of obesity and overweight was 5.39% and 22.72% among adults aged ≥18 years in 2002 [25], which rose to 7.74% and 29.87%, respectively, in 2010 [26]. Besides, trends in other factors of T2DM, such as decreasing physical activity levels and dietary changes, may be proposed explanations for the rise in diabetes [23]. However, Chinese national surveys showed that the proportion of adults meeting the minimum leisure-time physical activity increased over time (17.2% in 2002, 18.1% in 2005, and 22.8% in 2014) [24] and the average total dietary fiber intake in adults remained at a stable level during 1991-2011 [27]. Except for the contributions of the diabetes-related factors, the increase in diabetes may also be attributed to the efficiency change of the diabetes surveillance system. In 2009, we began to use the completely computerized diabetes surveillance system; with more convenient case-reporting procedure and stricter quality control measures, the rate of missing reports was diminishing. Meanwhile, the National Basic Public Health Services Project in China was begun in 2009. The project required that a certain proportion of T2DM cases in the districts must be found, reported, and followed up by the CDCs and local health services centers, which, to some extent, could increase the possibility of being diagnosed among undiagnosed diabetes cases.

In particular, the T2DM incidence also showed a decreasing pattern after 2014, which has been seen in Hong Kong (2007-2014) [8], Korea (2009-2011) [28], USA (2008-2012) [20], Denmark (2005-2007) [22], and Scotland (2005-2013) [14]. The decrease in the T2DM incidence after 2014 may be due not only to a real decrease in disease incidence, but also to the decreased prevalence of adulthood obesity and a reduction in the pool of undiagnosed diabetes [8, 14, 22]. Nevertheless, without firm data to support these hypotheses, our observations of the decreasing T2DM incidence over the last 3 years during the study period should be viewed with caution.

The sex and age disparities in diabetes incidence have been observed in previous reports [ 8, 15, 29]. In accord with these studies, we showed higher incidence rates of T2DM in male and older adults. However, the incidence increased more rapidly in male sex and younger age, again in keeping with the findings reported in Canada [30]. These different patterns may be explained by the higher prevalence of overweight/obesity in men and older adults and the greater rise in men and younger adults [24]. Besides, we found that the incidence rate of T2DM was slightly higher in urban areas than in rural areas, consistent with the findings in another Chinese population-based study in Harbin [7]. However, importantly, our analysis showed a greater average annual increase in incidence rate in rural areas (5.74%) than in urban areas (1.17%), which suggested a rapid growth of diabetes in rural areas over the last decade, and necessary intervention measures must be implemented.

The present study had several strengths. This study was one of the few population-based studies examining the T2DM incidence and time trends in mainland China. The analyzed database was extracted from the computerized diabetes surveillance system, which was continually able to monitor diabetes incidence rate among a province-wide population. With a large sample of 879,769 incident T2DM cases diagnosed by certificated health practitioners, the incidence and time trends of diabetes during 2007 and 2017 were reported eventually.

Our study had some limitations. First, we have begun to conduct an ongoing validation study on the registered diabetes cases since 2016. During the test, a certain number of diabetes cases were randomly selected, and a series of core diagnostic information, including medical page, as well as prescription and laboratory results of beta-cell autoantibodies, fasting/random blood glucose, glycosylated hemoglobin, routine urine (ketone body), etc., was collected and reviewed by an invited endocrinologist. If the endocrinologist concluded that the information collected supported the current case of diagnosed diabetes, we considered that the diagnosed case of diabetes was consistent. According to the analyses, we found that the consistency proportion in the validation study ranged from 86.07% to 95.98% during 2016-2018, indicating that our reported incidence was exaggerated anyway. Besides, we also conducted the underreporting investigations and calculated the underreporting proportion [31], which usually would underestimate the reported diabetes incidence. The underreporting proportion of diabetes was calculated as diagnosed but not reported cases divided by the total diagnosed cases in the investigation and multiplied by one hundred percent. Based on the underreporting investigations conducted in 2007 and 2016, the underreporting proportion of diabetes was reported to be 45.07% and 22.05%, respectively. Compared with the consistency proportion, we speculated that our reported diabetes incidence might be underestimated. Besides, despite lacking specific data, admittedly, the underdiagnosing proportion of diabetes in old people (e.g., age group ≥ 80 years) is usually high due to the fact that they are inconvenient to move and tend to not go to a doctor. To some extent, this would also underestimate our reported diabetes incidence. Second, in multivariable Poisson regression models, we only adjusted for sex, age, residence area, and calendar year, while other diabetes-related factors including obesity status, physical activity level, and dietary behaviors were not considered in the analysis. Third, with a relatively short period of 11 years and limited cases from the population of Zhejiang province, it is difficult to make a long-term prediction of diabetes occurrence in the study population and broader Chinese population.

In conclusion, this study suggested a significant increase in the incidence rate of T2DM among Chinese adults in Zhejiang province over the past decade, especially in adults characterized by male sex, younger age, and rural area.

## Figures and Tables

**Table 1:** Baseline characteristics of type 2 diabetes cases included in study, 2007-2017.

	Type 2 diabetes
Number of cases	879,769
Sex	
Males (%)	457,629 (52.02)
Females (%)	422,140 (47.98)
Age groups at diagnosis (years (%))	
20-29	12,304 (1.40)
30-39	44,904 (5.10)
40-49	138,391 (15.73)
50-59	240,761 (27.37)
60-69	236,792 (26.92)
70-79	142,871 (16.24)
≥80	63,746 (7.25)
Mean age at diagnosis (SD) (year)	59.61 (13.23)
Residence area	
Urban (%)	319,641 (36.33)
Rural (%)	560,128 (63.67)

**Table 2:** Mean incidence rate of type 2 diabetes, 2007-2017 (per 100,000 person-years).

Age at diagnosis (years)	Number of cases	Person-years	Incidence	95% CI
Males				
20-29	6,733	19,552,005	34.44	33.62-35.27
30-39	28,621	22,176,495	129.06	127.57-130.56
40-49	84,735	24,312,230	348.53	346.19-350.88
50-59	126,342	19,725,122	640.51	636.99-644.05
60-69	113,859	13,507,232	842.95	838.06-847.86
70-79	67,213	7,238,313	928.57	921.57-935.62
≥80	30,126	3,030,700	994.03	982.83-1005.32
≥20	457,629	109,542,097	417.77	416.56-418.98
Standardized incidence rate^∗^	718,216	244,966,073	293.19	292.51-293.87
Females				
20-29	5,571	19,282,194	28.89	28.14-29.66
30-39	16,283	22,360,608	72.82	71.71-73.95
40-49	53,656	24,157,195	222.11	220.24-224.00
50-59	114,419	18,950,805	603.77	600.28-607.28
60-69	122,933	13,066,014	940.86	935.61-946.14
70-79	75,658	7,349,879	1029.38	1022.06-1036.74
≥80	33,620	3,938,037	853.72	844.62-862.90
≥20	422,140	109,104,732	386.91	385.75-388.08
Standardized incidence rate^∗^	635,876	235,141,346	270.42	269.76-271.09
Overall				
20-29	12,304	38,834,199	31.68	31.13-32.25
30-39	44,904	44,537,102	100.82	99.89-101.76
40-49	138,391	48,469,425	285.52	284.02-287.03
50-59	240,761	38,675,928	622.51	620.02-625.00
60-69	236,792	26,573,246	891.09	887.51-894.69
70-79	142,871	14,588,192	979.36	974.29-984.45
≥80	63,746	6,968,737	914.74	907.66-921.87
≥20	879,769	218,646,829	402.37	401.53-403.21
Standardized incidence rate^∗^	1,352,605	480,107,419	281.73	281.26-282.20

^∗^Age-standardized to the 6th population census in Zhejiang Province, 2010. CI: confidence interval.

**Table 3:** Incidence rate ratios (IRR) of type 2 diabetes in relation to calendar year and demographic factors.

Characteristic	Males		Females		All	
	IRR	95% CI	IRR	95% CI	IRR	95% CI
Year						
2007	Ref.		Ref.		Ref.	
2008	1.165	1.137-1.194	1.148	1.122-1.176	1.157	1.137-1.176
2009	1.532	1.497-1.567	1.428	1.397-1.460	1.479	1.455-1.502
2010	1.864	1.823-1.905	1.713	1.677-1.750	1.786	1.758-1.813
2011	2.050	2.007-2.095	1.854	1.816-1.893	1.949	1.920-1.978
2012	2.138	2.094-2.184	1.892	1.853-1.932	2.011	1.981-2.040
2013	2.297	2.249-2.345	1.934	1.895-1.974	2.108	2.077-2.139
2014	2.326	2.278-2.375	1.913	1.874-1.952	2.110	2.080-2.141
2015	2.027	1.988-2.066	1.634	1.604-1.665	1.821	1.797-1.845
2016	1.870	1.835-1.906	1.462	1.434-1.489	1.657	1.635-1.679
2017	1.880	1.844-1.916	1.378	1.353-1.405	1.616	1.594-1.637
Age						
20-29 years	0.037	0.036-0.038	0.034	0.033-0.035	0.035	0.035-0.036
30-39 years	0.138	0.136-0.140	0.086	0.085-0.087	0.113	0.112-0.115
40-49 years	0.366	0.362-0.371	0.260	0.256-0.263	0.316	0.313-0.319
50-59 years	0.670	0.662-0.679	0.708	0.700-0.717	0.688	0.682-0.694
60-69 years	0.880	0.869-0.892	1.107	1.094-1.120	0.986	0.977-0.994
70-79 years	0.973	0.960-0.986	1.211	1.196-1.227	1.087	1.077-1.097
≥80 years	Ref.		Ref.		Ref.	
Sex						
Males					1.083	1.079-1.088
Females					Ref.	
Residence area						
Urban	1.079	1.072-1.085	0.929	0.923-0.935	1.005	1.001-1.009
Rural	Ref.		Ref.		Ref.	

Multivariable Poisson regression model with the calendar year as a dummy variable. IRR: incidence rate ratio; CI: confidence interval; Ref.: reference.

**Table 4:** Annual incidence rate of type 2 diabetes, 2007-2017 (per 100,000 person-years).

Characteristic	Year										
	2007	2008	2009	2010	2011	2012	2013	2014	2015	2016	2017
Males											
Number of cases	11,919	14,291	19,543	24,543	28,544	31,067	33,704	34,683	88,803	84,482	86,050
Person-years	6,151,185	6,221,090	6,289,684	6,375,636	6,578,491	6,702,012.5	6,665,077	6,686,235.5	19,211,085	19,269,113.5	19,392,488.5
Incidence	193.77	229.72	310.72	384.95	433.90	463.55	505.68	518.72	462.25	438.43	443.73
Standardized incidence rate^∗^	155.87	180.54	237.37	288.77	317.29	330.99	355.76	360.91	316.11	296.47	296.20
Females											
Number of cases	12,823	15,254	19,919	24,719	28,402	30,464	31,688	32,105	80,606	74,001	72,159
Person-years	6,023,793	6,111,332	6,219,344	6,304,557	6,511,975	6,660,241	6,643,162	6,700,593.5	19,167,958.5	19,283,995.5	19,477,780.5
Incidence	212.87	249.60	320.27	392.08	436.15	457.40	477.00	479.14	420.52	383.74	370.47
Standardized incidence rate^∗^	174.97	200.01	248.02	297.56	321.86	328.15	334.84	332.46	285.44	255.79	241.31
Overall											
Number of cases	24,742	29,545	39,462	49,262	56,946	61,531	65,392	66,788	169,409	158,483	158,209
Person-years	12,174,978	12,332,422	12,509,028	12,680,193	13,090,466	13,362,253.5	13,308,239	13,386,829	38,379,043.5	38,553,109	38,870,269
Incidence	203.22	239.57	315.47	388.50	435.02	460.48	491.36	498.91	441.41	411.08	407.02
Standardized incidence rate^∗^	164.85	189.81	242.31	292.69	319.21	329.28	345.12	346.52	300.69	276.05	268.65
Males											
20-29 years	10.40	12.81	17.08	23.52	27.94	36.22	40.96	39.00	43.71	42.43	43.43
30-39 years	47.11	57.25	81.84	109.67	125.75	141.62	153.86	161.16	151.84	147.60	158.63
40-49 years	171.79	194.60	269.40	346.72	377.65	391.13	413.96	422.31	375.44	360.04	363.40
50-59 years	339.25	390.97	511.08	590.02	660.78	679.28	770.18	799.61	708.74	652.30	637.36
60-69 years	424.68	529.84	681.90	820.30	927.24	1013.13	1081.74	1085.91	901.14	824.55	805.52
70-79 years	641.43	716.03	879.53	1043.46	1111.61	1086.34	1089.58	1083.89	919.20	881.18	888.76
≥80 years	719.53	760.89	1031.83	1258.40	1216.61	1181.32	1239.86	1167.79	983.33	894.88	888.94
Females											
20-29 years	5.74	8.78	10.94	18.70	28.15	29.73	32.54	36.75	35.97	37.96	36.83
30-39 years	31.93	35.26	41.92	55.15	75.90	80.86	79.42	94.80	89.37	86.63	80.94
40-49 years	140.89	159.41	204.82	250.24	266.28	262.31	260.02	272.32	240.47	206.33	196.39
50-59 years	448.60	483.78	565.99	681.81	698.88	706.90	732.28	728.79	642.71	563.98	518.98
60-69 years	586.27	695.49	887.95	1036.62	1155.81	1230.23	1241.90	1180.22	980.42	866.82	802.68
70-79 years	676.70	804.79	1004.25	1213.43	1310.32	1276.60	1300.54	1244.55	1018.55	941.56	911.10
≥80 years	521.87	618.10	817.58	916.95	941.86	989.49	1073.24	1046.54	859.32	798.39	817.04

^∗^Age-standardized to the 6th population census in Zhejiang Province, 2010.

## Data Availability

The data used to support the findings of this study are available from the corresponding author upon request.

## Supplementary Materials

Supplementary Materials Supplementary Table 1: average annual percentage change of type 2 diabetes incidence by demographic factors.
