# Supplementary material for: Incidence and Time Trends of Type 2 Diabetes Mellitus among Adults in Zhejiang Province, China, 2007-2017
Source: J Diabetes Res. 2020 Jan 19;2020:2597953. doi: 10.1155/2020/2597953 (PMC6995322; doi:10.1155/2020/2597953)
Supplement: Supplementary Materials — Supplementary Table 1: average annual percentage change of type 2 diabetes incidence by demographic factors. [file 2597953.f1.pdf]

Supplementary Table 1.Average annual percentage change of type 2 diabetes incidence by demographic factors

Characteristic	Average annual percentage change (%)	95% CIs	
		Lower (%)	Upper (%)
Males			
20-29 years	12.07	11.12	13.02
30-39 years	8.95	8.53	9.37
40-49 years	4.31	4.08	4.54
50-59 years	3.71	3.52	3.91
60-69 years	2.48	2.28	2.68
70-79 years	0.61	0.37	0.86
≧80 years	-1.41	-1.78	-1.03
≧20 years	5.19	5.09	5.30
Females			
20-29 years	13.88	12.80	14.96
30-39 years	8.44	7.89	8.99
40-49 years	0.76	0.49	1.04
50-59 years	-0.33	-0.51	-0.14
60-69 years	-0.28	-0.47	-0.10
70-79 years	-0.24	-0.46	-0.01
≧80 years	0.76	0.39	1.12
≧20 years	2.79	2.69	2.89
Residence area			
Urban	1.17	1.06	1.28
Rural	5.74	5.64	5.83
Overall			
20-29 years	12.89	12.18	13.60
30-39 years	8.72	8.39	9.06
40-49 years	2.84	2.66	3.02
50-59 years	1.73	1.59	1.86
60-69 years	1.04	0.90	1.18
70-79 years	0.14	-0.02	0.31
≧80 years	-0.22	-0.48	0.04
≧20 years	4.01	3.94	4.08

Multivariable Poisson regression models with the calendar year as a continuous variable and adjusting for the other covariates.

CI: confidence interval
